# Active surveillance versus initial surgery in the long-term management of Bosniak IIF–IV cystic renal masses

**DOI:** 10.1038/s41598-022-14056-6

**Published:** 2022-06-17

**Authors:** Lassi Luomala, Juhana Rautiola, Petrus Järvinen, Tuomas Mirtti, Harry Nisén

**Affiliations:** 1 Department of Urology, Helsinki University Hospital and University of Helsinki, Helsinki, Finland; 2 HUSLAB Laboratory Services and Research Program in Systemic Oncology, Diagnostic Center, Helsinki University Hospital and University of Helsinki, Helsinki, Finland; 3 Research Program in Systemic Oncology, University of Helsinki, Helsinki, Finland

**Keywords:** Renal cell carcinoma, Surgical oncology

## Abstract

There may be surgical overtreatment of complex cystic renal masses (CRM). Growing evidence supports active surveillance (AS) for the management for Bosniak IIF–III CRMs. We aimed to evaluate and compare oncological and pathological outcomes of Bosniak IIF–IV CRMs treated by initial surgery (IS) or AS. We identified retrospectively 532 patients with CRM counseled during 2006–2017. IS and AS were delivered to, respectively, 1 and 286 patients in Bosniak IIF, to 54 and 85 patients in III and to 85 and 21 patients in Bosniak IV. Median follow-up was 66 months (IQR 50–96). Metastatic progression occurred for 1 (0.3%) AS patient in Bosniak IIF, 1 IS (1.8%) and 1 AS (1.2%) patient in Bosniak III and 5 IS (3.5%) patients in Bosniak IV, respectively. Overall 5-year metastasis-free survival was 98.9% and cancer-specific survival was 99.6% without statistically significant difference between IS and AS in Bosniak IIF–IV categories. AS did not increase the risk of metastatic spread or cancer-specific mortality in patients with Bosniak IIF–IV. Our data indicate AS in Bosniak IIF and III is safe. Surgery is the primary treatment for Bosniak IV due to its high malignancy rate.

## Introduction

The reported incidence of complex cystic renal masses (CRM) and renal cell carcinoma (RCC) have increased over the last decades mainly due to the frequent use of abdominal imaging^1^ and observing small renal masses (SRM) which are < 2 cm in size^2^. Up to 15% of RCC may have a cystic component^3^. In 1986, Dr. Morton Bosniak, introduced the first radiographic classification that assessed the risk of malignancy CRMs—the Bosniak classification^4^. The classification and its updated versions^5^, have been the cornerstone of distinguishing potentially malignant CRMs from their benign counterparts. The classification was based on contrast-enhanced computed tomography (CT), which is used to identify the thickness and nodularity of cystic walls and septa. According to the 2019 update, magnetic resonance imaging (MRI) may also be used in the classification^6^.

**Figure 1:**
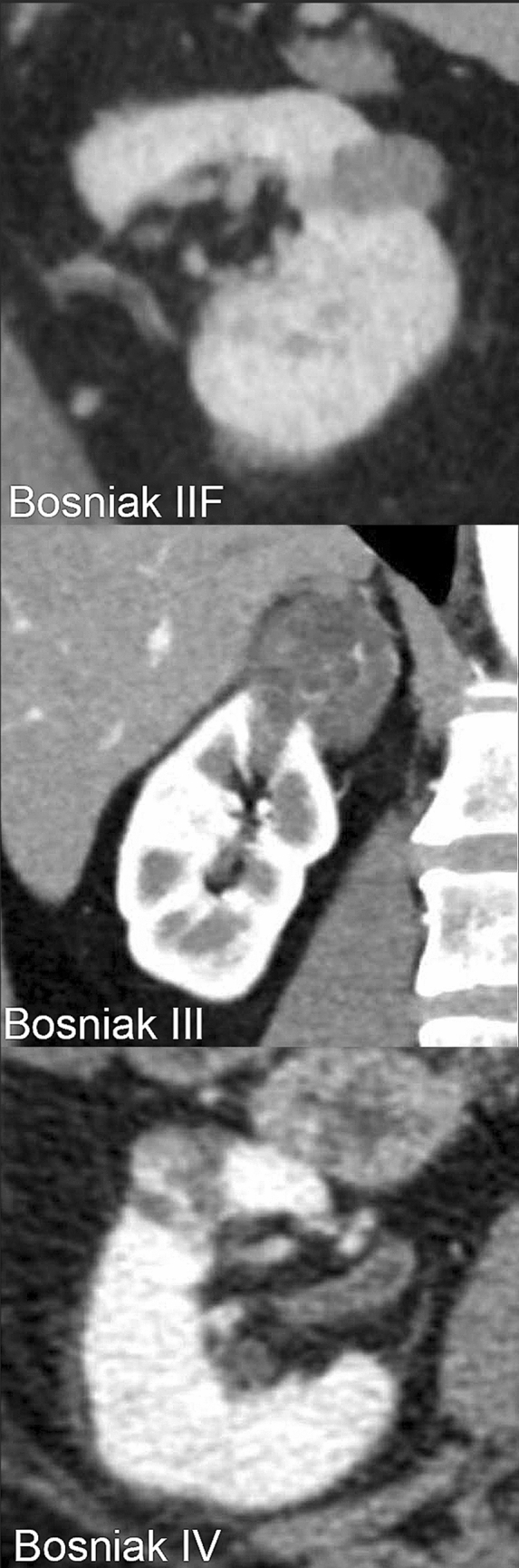
Cystic renal masses of Bosniak classification IIF, III and IV.

Bosniak I and II are considered as benign, whereas categories III and IV have a clinically relevant risk for malignancy. Bosniak IIF CRMs are usually benign but require radiographic follow-up. Traditionally, partial nephrectomy or radical nephrectomy has been recommended as initial management for Bosniak III and IV CRMs^7–9^. Figure 1 shows examples of CRMs of Bosniak IIF, III and IV.

The increased number of surgical interventions in the treatment of renal masses, mainly for the SRMs, has not decreased the mortality due to RCC which has stirred up discussion about the use of active surveillance (AS)^10^. Recently, the American Urological Association (AUA) guidelines added the option of AS in the management of Bosniak III and IV CRMs, especially those of < 2 cm in size^8^. Similarly, Bosniak classification version 2019^6^ and the European Association of Urology (EAU) guidelines 2020^9^ recommend either surgery or AS for patients with Bosniak III CRM. Thus, the counselling of patients with Bosniak III CRM is largely based on patient and surgeon preferences.

The risk of RCC varies between Bosniak categories. According to a meta-analysis by Schoots et al.^10^, RCC incidence was 6–18% for Bosniak IIF, 51–55% for Bosniak III and 89–91% for Bosniak IV CRMs. In the same study, metastases were found in 0.8% of the patients with Bosniak III/IV during a follow-up of 31 months. In addition, some recent reports showed no metastatic progression in patients with Bosniak III CRMs who were operated or were on AS^11–13^.

Furthermore, cystic RCC is frequently of lower Fuhrman grade and shows better survival than solid clear cell RCC of the same clinical stage^10^. These data indicate that there may be surgical overtreatment especially in patients with Bosniak III CRMs. Unfortunately, there is few reported data for AS in patients with Bosniak III CRMs.

The current treatment recommendations are based on retrospective studies with small cohorts and short follow-up. The lack of prospective studies therefore necessitates retrospective studies with larger cohorts. The aim of the study was to evaluate the management, pathological and oncological outcomes of patients with Bosniak IIF–IV CRMs in a large single center series.

## Patients and methods

### Study cohort

A retrospective single-center study of patients with CRMs was conducted in Helsinki University Hospital (HUH). Ethical approval was obtained from the Institutional Review Board (The Ethical Committee of Helsinki University Hospital, diary number HUS/1040/2018) and the hospital study permit from the corresponding unit head (HUS/419/2018). The study was conducted following Good Clinical Practice (GCP) guidelines in accordance with the ethical principles that adhere to the tenets of the Declaration of Helsinki. The Ethical Committee of Helsinki University Hospital waived the need to obtain informed consent for this registry-based study, since the national legislation allows the use of patient data with the above permits for research purposes without informed consent.

**Figure 2:**
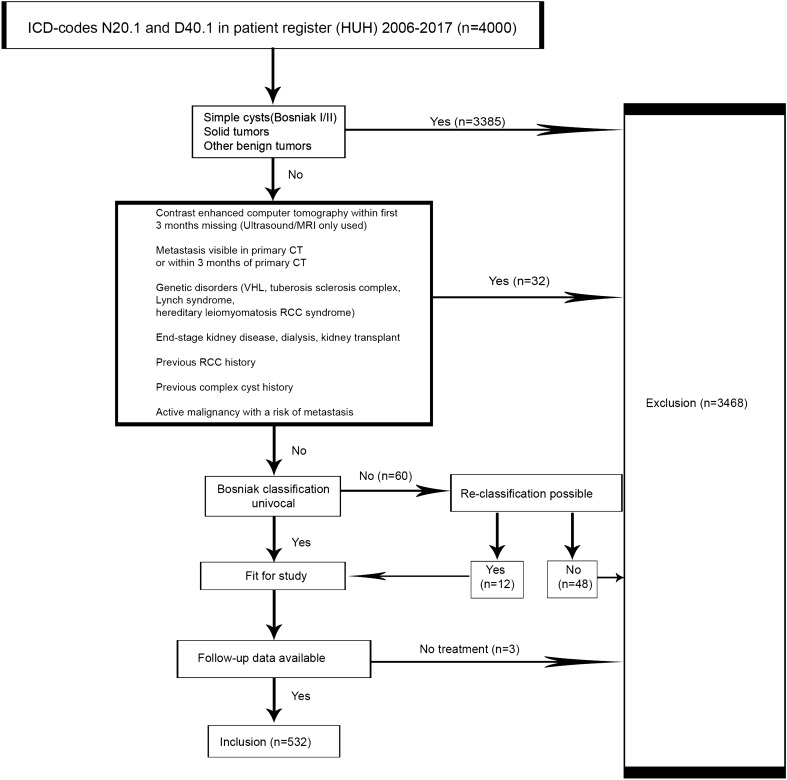
Patient flow chart.

We searched the patient register of HUH with ICD-10 codes N28.1 and D41.0 to find all patients (≥ 18 years) with CRMs or unspecified renal tumors counseled between January 1, 2006 and December 31, 2017 in the HUH area. Demographic characteristics were collected by patient chart review. All imaging charts were reviewed to identify CRMs of Bosniak IIF, III and IV interpreted by tertiary referral hospital uro-radiologists. The Bosniak classifications were based on 3 or 4-phase contrast enhanced CT-imaging and MRI and ultrasound were regarded as additional imaging tools^3,4^. If several CRMs were encountered, then the index CRM with the highest Bosniak category and the largest diameter was chosen for analysis. Exclusion criteria are detailed in the patient flow chart (Fig. 2). A total of 60 patients had a CRM in imaging but lacked Bosniak classification. Twelve of these patients were re-classified and 48 were excluded due to the lack of reliable imaging results. Three patients were excluded because they were not operated initially nor actively surveilled due to patient-related reasons.

In addition to serving as a national referral center HUH is responsible for delivering urological care for the whole Helsinki capital region. Except a small private sector, all urological counseling in the area happens at the HUH. The treatment and the length of surveillance were decided by attending urologists and the decisions were frequently discussed at multidisciplinary meetings of urologists and radiologists. Imaging intervals during AS varied from 3 to 12 months up to 3–5 years and continued variously thereafter. The shared information system allowed monitoring the patient status. Patients who moved away from the HUH administrative area during follow-up were surveilled until the last clinical appointment.

### Variables and definitions

Initial surgery (IS) refers to the surgical treatment of a CRM within five months of radiographic diagnosis. AS refers to patients who did not undergo IS but were followed with recurrent imaging to identify relevant changes in the CRM structure. Delayed surgery (DS) refers to surgical treatment of the CRM during AS. Concerning surgical pathology, histological type, 4-tiered International Society of Urologic Pathologists (ISUP)/World Health Organization (WHO) grade and TNM-staging were recorded and adapted to the 2018 version. Patient status (alive, metastatic or dead) and possible cause of death by the time of the cut-off date (July 10, 2019) were confirmed using medical records or Statistics Finland’s statistical database. Follow-up was defined as the time in months between the initial diagnostic abdominal imaging to the date of the last clinical verification of patient status or date of metastasis or patient death. Survival refers to the time from diagnosis to signs of metastasis (Metastatic free survival, MFS) or to RCC related death (Cancer specific survival, CSS) or until the end of data collection (cut-off date).

### Study end points and analysis

The primary end point of the study was to evaluate MFS and CSS of Bosniak IIF, III and IV CRMs. Secondary endpoints were malignancy rates of patients undergoing IS and DS and radiographic upgrading of Bosniak category. Comparisons between patients in IS and AS were performed in groups stratified by Bosniak classification.

### Statistics

Descriptive statistics included median (interquartile range, IQR) of continuous variables and frequency (percentages) of categorical variables. Continuous variables were compared by the Kruskal–Wallis test and pair-wise comparisons were made by the Mann–Whitney-U test. Categorical variables were compared by the Fisher exact test. Survival analyses were obtained by Kaplan–Meier analysis and compared by the Log rank test. A *p* < 0.05 was considered significant. All analyses were performed with IBM SPSS (version 26).

## Results

The study cohort comprised 532 patients with CRMs of Bosniak categories IIF–IV. Clinical characteristics of patients and histology of the removed CRMs are detailed in Table 1. No statistically significant differences were found for age or sex between the Bosniak categories. Bosniak IV masses were larger than those of other categories (*p* < 0.001). Biopsies were taken more often in the Bosniak III category compared to the other categories (*p* < 0.001). The rates of IS in Bosniak IIF, III and IV categories were 0.3%, 39% and 80%, respectively. The most common malignant histological subtypes of the operated patients were clear cell carcinoma (68%) and papillary carcinoma (22.6%). Partial nephrectomy was selected as the surgical modality in most of the cases (65%) as detailed in Table 1. Patients in the AS group were significantly older than in the IS group (mean 64.2 vs 60.7, *p* = 0.010).

**Table 1:** Clinical characteristics of patients and histology of CRMs by Bosniak stratification.

	Bosniak IIF	Bosniak III	Bosniak IV	*P* value
Patients, n/N (% of total)	287/532 (53.8)	139/532 (26.1)	106/532 (19.9)	
Age at diagnosis, mean, years ± SD	63 ± 13.3	64 ± 13.6	63 ± 14.0	0.597
**Sex, n (% of Bosniak class)**				0.666
Male	182/287 (63.4)	82/139 (58.9)	62/106 (58.5)	
Female	107/287 (36.6)	57/139 (41.0)	44/106 (41.5)	
Size of cystic mass, median, cm (IQR)	2.60 (1.5–4.8)	2.90 (2.0–5.1)	4.3 (2.8–6.1)	< 0.001*
Renal mass biopsy, n (% of Bosniak class)	9 (3.1)	18 (12.9)	7 (6.6)	< 0.001
Follow-up time, median (IQR), months	64 (51–88)	62 (48–104)	73 (47–105)	0.442**
Initial surgery, IS, n/N (% of Bosniak class)	1/287 (0.3)	54/139 (38.8)	85/106 (80.2)	
**Active surveillance, AS**	286/287 (99.7)	85/139 (61.2)	21/106 (19.8)	< 0.001
Surveillance only	276/287 (96.1)	67/139 (48.2)	6/106 (5.7)	
Delayed surgery, DS	11/287 (3.8)	18/139 (12.9)	15/106 (14.2)	
**Surgical treatment, IS + DS**	12/287 (4.2)	72/139 (51.1)	100/106 (94.3)	< 0.001
Radical nephrectomy	1/287	18/139	45/106	
Partial nephrectomy	11/287	54/139	55/106	
Malignant histology of operated CRMs, n/N (% of Bosniak class)	5/12 (41.7)	56/72 (77.8)	88/100 (88.0)	0.014 (IIF vs III), 0.001 (IIF vs IV), 0.062 (III vs IV)
**Histological subtypes, n (% of malignant histology)**				0.261
ccRCC	3 (60)	35 (62.5)	63 (71.6)	
**Papillary RCC**	2 (40)	11 (19.6)	21 (23.9)	
Type I	2	5	7	
Type II	0	2	6	
Unspecified	0	4	8	
Multilocular cystic RCC	0	6 (10.7)	2 (2.3)	
Chromofobe RCC	0	3 (5.3)	2	
Translocation carcinoma	0	1 (1.7)	0	

*CRM* complex cystic renal mass, *ccRCC* clear cell renal cell carcinoma, *RCC* renal cell carcinoma.

*P* values were calculated using the Kruskall–Wallis and Fisher exact test.

*IIF versus III *p* = 0.447, IIF versus IV *p* < 0.001, III versus IV *p* < 0.001.

**IIF versus III *p* = 0.684, IIF versus IV *p* = 0.107, III versus IV *p* = 0.428.

### Bosniak IIF

AS was delivered to 286 (99.7%) patients and DS was performed on 11 (3.8%) patients on AS at median time to intervention of 18.7 months (IQR 8–24). Malignancy in surgical pathology was found in 41.6% (5/12) of patients for IS and DS combined and 0% (0/1) and 45.4% (5/11) in IS and DS, respectively (*p* = 0.056). In all, 18 of 286 (6.3%) Bosniak IIF CRMs on AS were upgraded to Bosniak III, IV or solid tumor and 268 masses (93.7%) remained stable or downgraded. Within the upgraded cases, the median time to upgrading was 31.5 months (IQR 13–55). Ten of the upgraded masses were operated and five (50.0%) were malignant.

The median follow-up of IS and AS combined was 65 months (IQR 51–88). Metastatic progression occurred in none of the patients in IS and one patient in AS. This patient was alive and in oncological treatment at the time of the cut-off date (see supplementary material). The 5-year MFS was 100% for IS and 99.5% for AS (Log rank *p* = 0.944). Cancer-related deaths did not occur in Bosniak IIF category.

### Bosniak III

AS was delivered to 85 of 139 patients (64.7%) and DS was performed in 18 (21.2%) patients on AS at a median time to intervention of 12 months (IQR 7–26). Malignancy in surgical pathology was found in 77.8% (56/72) of operated patients in total of which 75.9% (41/54) and 83.3% (15/18) were IS and DS patients, respectively (*p* = 0.535). A radiographic upgrading to Bosniak IV or solid tumor was recorded in six AS patients (7.1%) of which two were operated and both appeared malign (100%). Median time to upgrading was 47 months (IQR 10–77).

The median follow-up of IS and AS combined was 63 months (IQR 49–105) with no statistical difference between IS and AS. Metastatic progression occurred in one (1.8%) patient in IS, and one patient (1.2%) on AS. The 5-year MFS was 98.1% for IS and 98.7% for AS (Log rank *p* = 0.755; HR 1.548; 95% CI 0.097–24.750). No patients in IS and one patient on AS died of RCC during follow-up. The 5-year CSS was 99.3% in total and 100% and 98.6% for IS and AS, respectively (Log rank *p* = 0.438).

### Bosniak IV

IS was performed in 85 (80.1%) of 106 patients and the rest (19.8%) were on AS. DS was performed on 15 (71.4%) of 21 AS patients with median time to intervention of 19 months (IQR 10–50). Malignancy in surgical pathology was found in 88% (88/100) of patients in total and 88.2% (75/85) and 86.7% (13/15) of IS and DS patients, respectively (*p* = 0.535).

The median follow-up of IS and AS combined was 76 months (IQR 48–105). Metastatic progression developed in five patients (5.9%) in IS and none on AS. The 5-year MFS was 97.5% and 100.0% for IS and AS (Log rank *p* = 0.265). Two patients given IS died of RCC. The 5-year CSS was 99.0% in total and 98.8% and 100.0% for IS and AS, respectively (Log rank *p* = 0.466).

### The whole cohort

Overall, metastatic progression developed in 8 of 532 patients (1.5%) and the 5-year MFS was 98.9%. The median follow-up was 66 months (IQR 50–96) for the whole cohort and 77 months (IQR 49–105) for IS and 64 months (IQR 51–91) for AS (*p* = 0.211). Case reports of metastasized patients are detailed in the supplementary material.

In total, 3 of 532 (0.6%) patients died of RCC during follow-up and the 5-year CSS was 99.6% overall and 98.5% among patients with pathologically confirmed RCC. We did not find significant differences comparing MFS or CSS by Log rank test between IS and AS groups stratified by Bosniak category.

## Discussion

We present a large cohort of patients with CRMs managed at our hospital. The observed treatments reflect good adherence to modern guidelines^8,9^. The oncological results were favorable for all Bosniak categories. However, we found eight patients (1.5%) with metastatic progression after the diagnosis of a CRM and three of them died because of cancer progression. Most authors have reported zero or very few metastatic progressions in patients with Bosniak IIF and III^10,12–15^, whereas our data indicate low but not negligible levels of metastatic potential in CRMs. Importantly, the length of surveillance in many of the reported studies was from 2–4 years^10,13–15^, whereas we had the longer follow-up time of 5.3 years in our study. Regarding the slow growth and progression times of cystic RCC^14,16^, the reported follow-up times may still be too short to catch all metastatic events. Renal tumor biopsy has become an essential tool in the diagnosis of suspect solid renal masses. Needle biopsy of a cystic lesion is not recommended because of low sensitivity and fear of tumor spillage. However, the nodular component of Bosniak IV may be successfully targeted via solid renal parenchyma^8,9^.

Age, frailty and comorbidity may strongly influence the treatment selection of renal complicated masses. In our data the patients on AS were 3.5 years older than those in IS. They probably also had more comorbidities but we did not have enough data from AS to show this. Renal simple cysts and CRMs are more frequent in patients with renal insufficiency. In end stage renal disease, while patients are waiting for kidney transplant, kidneys with suspicious CRMs are an indication for nephrectomy. In our study group these patients were excluded. Surgical complexity of the CRM and patient attitude on surgery and surveillance may certainly play a major role.

In the Bosniak IIF category, 99.7% of the patients were assigned to AS. Radiographic upgrading was recorded in 6.3% of Bosniak IIF cases and 50% of these were malignant. In other studies^10,17^, upgrading of the Bosniak IIF category was reported in 7–11% of the cases and malignancy was found in 78–85% of those upgraded. These high figures strongly support surgical treatment of upgraded Bosniak IIF CRMs, but we cannot estimate the true rate of malignancy in the whole Bosniak IIF category. Our data showed only 0.3% metastatic progression. This finding supports AS as the primary management of Bosniak IIF CRMs which is in line with the current EAU and AUA guidelines^8,9^.

In the Bosniak III category, 78.1% of the operated CRMs were malignant, which is higher than the 51% reported in a systematic review by Schoots et al.^10^. However, similar malignancy rates of 72–79% were also reported recently^16,18^. Interestingly, Sefik et al.^19^ showed that the malignancy rate was higher in Bosniak III CRMs with nodular features in the walls or septae compared to non-nodular (86.7% vs 54.1%, *p* = 0.026). Radiographic nodularity is also ranked as an important feature in the new Bosniak classification, version 2019^6^ and this might allow the adoption of new criteria when selecting patients for either IS or AS. In our study, DS led to a higher rate of malignancy (83%) compared to IS (76%) in surgical pathology. Unfortunately, there are few data on patients with upgraded Bosniak III CRMs. Given that radiographically upgraded Bosniak IIF CRMs show high malignancy in surgical pathology, the upgrading of Bosniak III could also indicate potential malignancy.

We identified 2 out of 139 patients (1.4%) with Bosniak III who had metastatic progression. One patient underwent IS and one started AS. These figures are comparable with 1 metastatic Bosniak III out of 373 patients in the systematic review of Schoots et al.^10^. Similarly, some studies show no metastatic progression in patients with Bosniak III^12–15^. Our results indicate that the occurrence of metastatic progression in patients with Bosniak III is uncommon but not negligible. Moreover, we show that IS does not give a clear advantage over AS with respect to metastatic progression or cancer-specific mortality.

In the Bosniak IV category, 94.3% of patients underwent either IS or DS and 88% of these were malignant. This is in line with previous studies^10,14,15,18^ that reported 85–90% of malignancy in Bosniak IV and confirms the category as a clinically valuable predictor of malignancy and thus, it necessitates a strong indication for surgery. However, Bosniak IV CRMs still showed excellent prognosis in our study as indicated by the low rate of metastasis and cancer related deaths.

Interestingly, all the metastasized CRMs were ≥ 2 cm in size in every category. This result may support the AUA guidelines^7^, which recommend AS as an option for initial management of SRMs and Bosniak III/IV CRMs, especially those of < 2 cm of size and for the patients whose life expectancy is low due to comorbidities.

Our study has some limitations along with being a retrospective and single-center series. First, selection between IS and AS was not controlled by a protocol but was based on the surgeon’s and patient’s preference. Second, we did not have a central review of radiographic assignments. Third, AS was not systematically organized after the first 3–5 years.

In conclusion, metastatic progression in Bosniak IIF–IV CRMs is uncommon. AS does not increase the risk of metastatic spread or cancer-specific mortality of patients with Bosniak IIF & III CRMs. Our results support the safety of AS in Bosniak IIF & III CRMs and confirms surgery as the primary initial treatment for patients with Bosniak IV due to its associated high malignancy rate. Moreover, the radiographic upgrading of Bosniak category in AS is a strong indicator of malignancy and surgical treatment is advisable. Randomized clinical trials between IS and AS in patients with Bosniak III CRMs are warranted. Longer follow-up schedules may be necessary to detect all metastatic events for patients on AS with CRMs.

## Supplementary Information

Supplementary Information.
