# Supplementary material for: Active surveillance versus initial surgery in the long-term management of Bosniak IIF–IV cystic renal masses
Source: Sci Rep. 2022 Jun 17;12:10184. doi: 10.1038/s41598-022-14056-6 (PMC9205856; doi:10.1038/s41598-022-14056-6)
Supplement: Supplementary file 1 — Supplementary Information. [file 41598_2022_14056_MOESM1_ESM.pdf]

Supplementary material

Case reports of metastasized patients

Patients with metastasized Bosniak IIF–V							
**Bosniak category**	**Diameter (cm)**	**Age (y)**	**Treatment (IS/AS, PN/RN)**	**MFS (mo)**	**CSS (mo)**	**Details**	**Surgical pathology**
IIF	2	49	AS(DS), PN	54	63 (alive)	Upgrading from Bosniak IIF to IV was recorded after 15 months (m) of AS and the patient underwent DS. The patient dropped out of regular post-operative follow-up after surgery due to patient related causes. Bone and visceral metastasis were found 54 m after the first imaging. Patient is alive and in oncological treatment	pT1a ccRCC grade II*, negative marginals
III	6	57	AS(DS), PN	31	43	After 12 m of AS wall enhancement and growth were seen and the patient underwent DS. After 31 m of primary imaging several lung metastases were detected and the patient died of RCC at 43 m after primary imaging despite oncological treatment.	pT3a papillary type I carcinoma, grade* I, negative marginals
III	2.1	61	IS, PN	15	25(alive)	A solitary lung metastasis was found 15 m after the operation. Patient is alive and in oncological treatment.	pT1, ccRCC, grade* II, negative marginals
IV	5.3	80	IS, RN	13	13	Metastases were non-evident at the time of operation but were found in the liver 13 m after the operation when the patient was hospitalized during which the patient died of RCC.	pT1b, ccRCC, grade* II
IV	9	48	IS, RN	118	121	Lung and adrenal metastasis were found 118 mo after the operation. The patient is alive and in oncological treatment	pT1b, ccRCC, grade* II
IV	11	65	IS, RN	75	92	A solitary metastasis in the contralateral adrenal gland was found 75 months after primary operation and was treated by adrenalectomy. Multiple metastases in contralateral kidney and pancreas were later observed and cytoreductive nephrectomy was performed. Patient is alive and in oncological treatment.	pT2b ccRCC, grade* II
IV	17	67	IS, PN	38	92	Several cystic masses were seen in both kidneys before operation. The largest cystic mass was operated by PN and positive marginal were reported. Another contralateral	pT2b, papillary type I carcinoma, grade* II,

						cystic mass (also Bosniak IV) was operated 7 mo later by RN due to growth and increased enhancement. Both tumors were reported as papillary carcinoma in pathology report. Nodal and retroperitoneal metastases were found 38 months after the first operation. The patient died of RCC at 92 months after the first operation.	positive marginals
IV	25	55	IS, PN	65	81	Small 15 mm tumor was found in the contralateral kidney 65 months after primary operation and was treated by PN in a curative manner. Chromofobe RCC was reported in surgical pathology of this contralateral metastasis. The patient is alive without signs of progression.	pT3a, ccRCC, grade* III, negative marginals

* International Society of Urologic Pathologists (ISUP)/World Health Organization (WHO) grade
IS = Initial surgery
AS = Active surveillance
DS = Delayed surgery
RCC = Renal cell carcinoma
ccRCC = clear cell renal cell carcinoma
RN = Radical Nephrectomy
PN = Partial nephrectomy
mo = months
